# Models of Individual Dietary Behavior Based on Smartphone Data: The Influence of Routine, Physical Activity, Emotion, and Food Environment

**DOI:** 10.1371/journal.pone.0153085

**Published:** 2016-04-06

**Authors:** Edmund Seto, Jenna Hua, Lemuel Wu, Victor Shia, Sue Eom, May Wang, Yan Li

**Affiliations:** 1 Environmental & Occupational Health Sciences, School of Public Health, University of Washington, Seattle, Washington, United States of America; 2 Environmental Health Sciences, School of Public Health, University of California, Berkeley, California, United States of America; 3 Electrical Engineering and Computer Science, School of Engineering, University of California, Berkeley, California, United States of America; 4 Public Health Nutrition, School of Public Health, Seoul National University, Seoul, South Korea; 5 Community Health Sciences, Fielding School of Public Health, University of California Los Angeles, Los Angeles, California, United States of America; 6 Maternal and Child Health, School of Public Health, Kunming Medical University, Kunming, China; University of North Carolina at Charlotte, UNITED STATES

## Abstract

**Introduction:**

Smartphone applications (apps) facilitate the collection of data on multiple aspects of behavior that are useful for characterizing baseline patterns and for monitoring progress in interventions aimed at promoting healthier lifestyles. Individual-based models can be used to examine whether behavior, such as diet, corresponds to certain typological patterns. The objectives of this paper are to demonstrate individual-based modeling methods relevant to a person’s eating behavior, and the value of such approach compared to typical regression models.

**Method:**

Using a mobile app, 2 weeks of physical activity and ecological momentary assessment (EMA) data, and 6 days of diet data were collected from 12 university students recruited from a university in Kunming, a rapidly developing city in southwest China. Phone GPS data were collected for the entire 2-week period, from which exposure to various food environments along each subject’s activity space was determined. Physical activity was measured using phone accelerometry. Mobile phone EMA was used to assess self-reported emotion/feelings. The portion size of meals and food groups was determined from voice-annotated videos of meals. Individual-based regression models were used to characterize subjects as following one of 4 diet typologies: those with a routine portion sizes determined by time of day, those with portion sizes that balance physical activity (energy balance), those with portion sizes influenced by emotion, and those with portion sizes associated with food environments.

**Results:**

Ample compliance with the phone-based behavioral assessment was observed for all participants. Across all individuals, 868 consumed food items were recorded, with fruits, grains and dairy foods dominating the portion sizes. On average, 218 hours of accelerometry and 35 EMA responses were recorded for each participant. For some subjects, the routine model was able to explain up to 47% of the variation in portion sizes, and the energy balance model was able to explain over 88% of the variation in portion sizes. Across all our subjects, the food environment was an important predictor of eating patterns. Generally, grouping all subjects into a pooled model performed worse than modeling each individual separately.

**Conclusion:**

A typological modeling approach was useful in understanding individual dietary behaviors in our cohort. This approach may be applicable to the study of other human behaviors, particularly those that collect repeated measures on individuals, and those involving smartphone-based behavioral measurement.

## Introduction

Smartphone applications (apps) have made it possible to measure multiple aspects of a person’s behavior, including physical activity, diet, emotion, and time-location patterns. Some of these software apps leverage the ability of hardware sensors (such as accelerometers and GPS) to collect measurements with relative ease and minimal subject burden, over long periods of time. In recent years, personal monitoring has become popular among individuals who wish to quantify their own behavior, not only with smartphone apps, but also with a variety of personal monitoring devices[1, 2]. Researchers interested in behavior change potentially have much to learn from these individuals, not only because of the tendency for there to be large amounts of behavioral data available for these individuals, but because the process by which these individuals learn from their data can inform behavioral constructs, such as self awareness, self efficacy, and decisional balance. Studying these individuals may inform behavioral theories such as classical and operant conditioning[3] and theoretical frameworks such as Bronfenbrenner’s ecological framework for human development[4]. As more people use these personal devices and apps, there are opportunities to improve our understanding of how individuals learn from their monitoring data, recognizing that this learning is occurring naturally, outside of traditional psychological and cognitive science experimental settings.

Increasingly, studies of behavior change are recognizing the value of an “N of 1” approach, in which interventions are tailored to the individual [5]. This approach acknowledges subject uniqueness, and that interventions need to be effective first and foremost at the individual, rather than at the group level. The importance of tailoring interventions to the individual is gaining acceptance in other fields, e.g., personalized medicine [6], where studies of effectiveness of interventions at the individual level is a major departure from classic randomized clinical trials. Moreover, the “N of 1” approach is different from analytic approaches used in traditional epidemiology and public health. Such traditional approaches often identify associations between risk factors and outcomes, and are employed in large cohort studies to identify risk factors that are generalizable to the population being studied.

As a precursor to intervention studies aimed at assessing behavior change, methods are needed to characterize an individual’s baseline behaviors before interventions are introduced. This characterization includes understanding the regularity of an individual’s pattern of behavior, as well as either observable or hidden (latent) factors that might affect behavior. Such methods may also be of use to individuals who are self-monitoring as a way to synthesize and quantify the relationships between various behaviors they are tracking. Also, because no single behavioral model may be applicable to all persons, methods that allow for comparisons between alternative theories by fitting data to different models can be helpful in characterizing individuals.

This paper demonstrates individual-based modeling methods relevant to a person’s eating behavior. Behavioral data were collected using a smartphone app that tracks diet, physical activity, emotion, and time-location patterns. Using these data, we evaluated four alternative hypotheses that potentially explain an individual’s eating behavior–“typologies” of eating. The first hypothesis is that an individual follows a “routine”, and that time of day is a good predictor of the type of meal this person will consume. The second hypothesis is that an individual follows an “energy balance” model, in which the person’s diet is responsive to physical activity energy expenditure. The third hypothesis is that an individual follows an “emotional” model, and that the person’s affective state alters eating behavior. The fourth hypothesis is that an individual is influenced by the surrounding “food environment”, and a combination of this individual’s GPS mobility tracking data and GIS food establishment data can be used to model the person’s diet. To illustrate the value of an individual-based modeling approach, we compared the performance of individual-based models to a typical regression modeling approach in which a multivariate explanatory model was used to fit the pooled data from all monitored subjects.

By using mobile monitoring data to explore the different typologies described above we aim to address some of the complexity presented in previous conceptual models that describe the multitude of factors that affect obesity, which include unhealthy individual behaviors as well as environmental characteristics that may influence an individual’s behavior [7]. While conceptual models and frameworks are useful in clarifying the linkages between various factors associated with obesity (e.g., the EnRG framework [8], the IoTF model [9] and SPOTLIGHT model [10]), our paper aims to illustrate how quantitative modeling approaches may be used to examine factors that affect an individual’s diet patterns.

## Methods

### CalFit Chi and Dong Smartphone Application

We developed a software application called CalFit Chi and Dong (Chi means “to eat” and Dong means “to move” in Chinese) that runs on Android smartphones, and tracks diet, physical activity, emotion, and time-location patterns. For physical activity assessment, the app records 3-axis accelerometry data at 10 Hz, from which energy expenditure is computed using algorithms previously developed by our group [11, 12]. For time-location patterns, the app logs GPS data at 10-second intervals, from which the activity space of the phone’s user can be mapped. To understand emotion, the app implements Ecological Momentary Assessment (EMA) [13, 14]–the phone rings during five times throughout the day. Each time the phone rings, it prompts the user to complete a short survey on the user’s phone. A total of six questions are asked in the EMA survey (5-choice Likert ratings of happiness, stress, tiredness, and sadness; and two questions about where and with whom previous meals were consumed). On Samsung Galaxy Y phones, with no other apps running, the app can run for approximately 20 hours on a single battery charge. Data are encrypted and stored locally in the memory on the phone.

Additionally, the phone is used to assess diet. Study subjects are asked to use the video camera on their smartphones to record a voice-annotated video of each meal and snack they consume. Our approach is one of a number of emerging mobile phone-based approaches to diet assessment[15, 16]. During the recording, subjects verbally describe the contents of their meal (ingredients and quantities of food), which is recorded with the video. Later, two trained dietitians familiar with local diets review the contents of the videos, and code the portion sizes and food groups associated with each food consumed. Subjects’ diet recordings were coded by both dietitians in order to assess inter-rater reliability. The coding accuracy of the dietitians was also assessed during training by having the dietitians code a previously prepared training video with known amounts of food items. All data are time-stamped by the clock on the phone, which allows the data to be merged.

### Study Cohort

This research was conducted as part of a larger ongoing study investigating patterns of urban change within Kunming and their associations with changing dietary behaviors and risk factors for obesity. Kunming is the capital city of Yunnan province in southwestern China. Like many medium-sized cities in China, Kunming is rapidly developing. Studies have documented increasing obesity trends, which have outpaced those in larger Chinese cities [17, 18]. For this larger ongoing study, we hypothesize that obesity-related factors such as dietary behavior are associated with the food environment, which is supported by evidence from studies conducted in the U.S. [19–21]. The U.S.-based studies are relevant to China, particularly because in recent years, western-style fast food has become increasingly prevalent and heavily advertised within medium-sized Chinese cities.

A convenience sample of 12 subjects was recruited from among students at the Kunming Medical University and monitored. All participants completed a basic demographic and quality of life questionnaire. Participants received hands-on training on the use of the CalFit Chi and Dong app. Each participant received a study phone, and asked to carry the smartphone for two consecutive weeks during all waking hours, except for periods of bathing or swimming. They were asked to carry the phone in a neoprene waist pouch to improve the quality of accelerometry measurement. Subjects were trained on standard portion sizes, and how to use CalFit apps on study phones. Aside from in-person training during recruitment, each participant also received a written protocol with detailed instructions and a handout with common food items with standard portion sizes as a reminder. No services were provided for study phones; however, participants received reminder text messages (charging the phone, record dietary data, etc.) on their personal phones. Physical activity and EMA were monitored continuously over two weeks. However to reduce participant fatigue, we limited diet recording to only 6 days (3 days in each week). After each week of monitoring, we retrieved the phones, and downloaded the data. In some cases, this resulted in a 1–2 day gap in monitoring between the two weeks. The Institutional Review Boards at the University of California Berkeley and Kunming Medical University approved our study protocols (protocol number 2012-05-4352) and consent procedures. Study participants provided their written informed consents to participate in this study.

### Data Analysis

Data from the phones were merged based on time-stamps. Because the different types of data were collected at different frequencies, we aggregated data as described below. In our study, our outcome is food portion size (in grams) consumed at each meal. Portion size is thought to be associated with obesity because it relates to latent variables such as hunger and satiety, which can change as a person ages, and possibly, because it is related to learned behaviors (e.g., taught to “clean your plate”) [22–24]. For each recorded meal, trained dietitians familiar with local diets reviewed the videos, and coded the portion sizes of each food group consumed. Inter-rater reliability of the dietitians was assessed by comparing different dietitians coded portion sizes for specific food groups using the Student’s t-test. We found no significant differences at the p<0.05 level between the dietitians. The coding accuracy was assessed by calculating the differences between coded and known amounts of food items in a prepared training video, as well as the percent errors. Similar to other studies [15, 25–29], on average, the differences between coded and known amounts of food items ranged between 8 to 20 grams, and percent errors ranged between 22 to 32%. To classify the timing of each person’s meals, we used cutoffs of < = 10 AM for “breakfast”, >10 AM to < = 2 PM for “lunch”, and >2 PM for “dinner”. Only one participant recorded one occasion of snacking, which was categorized as part of the meal according to the cutoffs.

Accelerometry data were converted to energy expenditure using an algorithm that corrects for the orientation of the phone, computes activity counts along the vertical axis and horizontal plane, and finally, converts the activity counts into energy expenditure [11, 12]. Energy expenditure was then aggregated into 1-hour averages for each person. We analyzed energy expenditure in kcals during the same hour as each meal, and kcals before each meal (the average kcals of three hours before the meal).

To assess emotion, we averaged the Likert scores for each EMA question collected multiple times per day for each person. We performed Principal Components Analysis (PCA) on the average daily results of three questions related to emotion (happiness, stress, and tiredness), which resulted in two major components that explained over 82% of the variation. The first component loaded heavily on happiness, while the second orthogonal component loaded on tiredness. This resulted in two components representing the average daily emotion reported by each person.

To assess each person’s exposure to different food environments, we used the GPS tracking data to look at the food environment within each person’s activity space. First, we processed the data using a “Staypoint” algorithm [30]. The algorithm filters the GPS data by identifying those places where an individual stays for more than a specified amount of time. We chose a threshold of 10 minutes as a reasonable trade-off between producing too many locations (many of which may be unimportant because the individual simply passes by these places), and not missing potentially important short stops within a person’s activity space (e.g., home, work, recreation, and the locations of transit stops and errands). For example, if a person spent 30 minutes at home, this would result in one Staypoint. In contrast, if a person is moving from one location to another, and does not spend more than 10 minutes at any given location, no Staypoint would be recorded.

In contrast to the U.S., for which various sources of GIS data for food environment analyses exist, food establishment data are difficult to obtain for China. For our study, we used Google map data, which offers a fairly comprehensive global database of establishments for cities, including Kunming. We developed a software application that automates the querying of food establishments using Google Places (https://developers.google.com/places). Google Places allows for searches on several keywords relevant to food establishments (bakery, bar, cafe, convenience store, food, grocery or supermarket, liquor store, meal delivery, meal takeaway, and restaurant). For each person’s Staypoints, we used Google Places to search for the numbers of establishments within a 0.25 km radius (easy walking distance from places a person spends time during their day) using each keyword. We divided the number of establishments by the number of Staypoints visited each day to obtain a daily average number of food establishments per Staypoint for each person.

We examined four alternative models behavior as described in Table 1. Each model consisted of a linear regression model, in which the dependent variable, portion size of a meal, was explained by one or more possible explanatory factors. In the first model (routine), the portion size of each person’s meal was estimated based on variables indicating whether the meal was a breakfast, lunch or dinner. In the second model (energy balance), portion size was estimated based on the energy expenditure at the same hour and the average of the 3 hours preceding the meal. In the third model (emotional), portion size was estimated based on the two principal components derived from the EMA questions. In the fourth model (food environment), portion size was estimated based on the average number of food establishments (of all types) encountered at each person’s Staypoints. Table 1 lists the conceptual basis for each of these models and some supporting evidence. For each of these models, we fit each person’s data separately, as well as fit the combined data from all persons. Additionally, for the combined data, we fit a full model, which included all explanatory variables from models 1–4. To further examine the association between food environment and food portion size, we ran separate full regression models using different types of food environment (e.g., bakeries, bars, cafes, etc.). Model goodness of fit was based on the coefficient of determination (R^2^). For the models that based on the combined data from all individuals, we computed overall R^2^ as well as the R^2^ for each specific individual within the combined dataset to allow for comparisons to the individual-based models’ coefficient of determinations. Finally, we also fit the data using a varying intercepts mixed effects model (lmer in lme4 for R), in which subject ID was a random effect in the model. The mixed effects model was specified similarly to the full model described before, with portion size as the outcome, and the timing of the meal, physical activity, EMA principal components, and the average number of food establishments per Staypoint as explanatory variables.

**Table 1:** Typological Models of Eating Behavior.

Model	Hypothesis	Conceptual Basis
Routine	Portion size of each person’s meal was estimated based on indicator variables indicating whether the meal was a breakfast, lunch or dinner	The concept of chronotypes—that there are time-based patterns in eating and obesity. e.g., Fleig and Randler used food logs and found differences in diet between morning vs. evening-oriented adolescents [31], and Schubert and Randler found that morningness was negatively associated with BMI in a cohort of German university students [32]
Energy Balance	Portion size was estimated based on the energy expenditure at the same hour and the average of the 3 hours preceding the meal	Exercise related to increase in carbohydrate and protein intake [33, 34]
Emotional	Portion size was estimated based on the two principal components derived from the EMA questions	Negative mood states may be associated with eating unhealthy foods [35]
Food Environment	Portion size was estimated based on the average number of food establishments encountered at each person’s Staypoints	Neighborhood food environment associated with unhealthy eating [19–21]

## Results

Descriptive statistics for the study population’s characteristics, including summaries of smartphone-derived behavioral measures are presented in Table 2. The average age of study participants was 24.6 years old with a standard deviation of 3.06, and ranged between 18 and 31 years old. The average body mass index (BMI) was 21.0 kg/m^2^ with a standard deviation of 3.69, and ranged between 17.0 to 30.5 kg/m^2^. Based on the World Health Organization BMI cutoff[36], among the 12 participants, two (17%) were underweight; two (17%) were overweight, and the rest were of normal weight (66%). From the questionnaire, all were happy with their then current lives, all reported having adequate sleep time with an average of 7.5 hours per night, and believed exercise is important in their life.

**Table 2:** Description of Study Subjects.

**Characteristics**	
Age, years (mean, SD, range)	24.6, 3.06, 18–31
Gender (% female)	66.7%
BMI, kg/m^2^ (mean, SD, range)	21.0, 3.69, 17.0–30.5
**Diet**	
Total portion size per meal, g (mean, SD, range)	284, 178, 5–1203
Dairy portion size per meal, g (mean, SD, range)	178, 70, 20–250
Protein portion size per meal, g (mean, SD, range)	65, 53, 5–350
Grain portion size per meal, g (mean, SD, range)	114, 82, 7–500
Vegetable portion size per meal, g (mean, SD, range)	84, 72, 5–350
Fruit portion size per meal, g (mean, SD, range)	196, 202, 10–1028
**Accelerometry**	
Hourly Energy Expenditure, kcal (mean, SD, range)	27, 17, 4.7–132
**Ecological Momentary Assessment**	
Happiness Score (0–4) (mean, SD, range)	2.3, 1.3, 0–4
Stress Score (0–4) (mean, SD, range)	0.2, 0.5, 0–4
Tiredness Score (0–4) (mean, SD, range)	0.72, 1.0, 0–4
Sadness Score (0–4) (mean, SD, range)	0.25, 0.70, 0–4
**Food Environment**	
Number of Staypoints per person (mean, SD, range)	33, 20, 6–78
Number of food establishments 0.25 km of Staypoints (mean, SD, range)	1158, 1102, 91–3829

Ample compliance with the phone-based behavioral assessment was observed for all participants. Across all individuals, 868 consumed food items were recorded. On average, subjects recorded 72 food items over the 6 days of diet assessment (the person who recorded the least, still recorded 56 food items). Generally, fruits, grains and dairy foods made up a larger share of their meals, while vegetables and proteins (including meat and beans) made up a relatively smaller portion of meals. Similarly, subjects complied with the use of the phones for accelerometry. On average, 218 hours of accelerometry were recorded for each participant. For the subject with the least monitoring, 180 hours of accelerometry were still collected. Finally, across all individuals, we recorded a total of 416 EMA responses and 35 responses on average per participant, with the least collected being 26 per participant. Participants on average recorded moderate levels of happiness, while stress, tiredness, and sadness were on average low. Responses to EMA questions spanned the entire range from scores of 0 to 4, indicating both variations between persons and between times for specific persons.

Based on the GPS data, we computed 400 Staypoints for all participants. On average there were 33 Staypoints per person, with 6 being the minimum. On average, each person encountered 1158 food establishments (based on a 0.25 km search centered on all of the person’s Staypoints) over the 2 weeks of tracking, or approximately 83 establishments per day. There was a fairly large range of variability in food environment exposures between participants. There was a problem with Subject 2’s GPS data, which did not allow us to assess their exposures to food environments. This individual was left in the analysis because of our low number of overall participants, and because the models that did not include food environment could still be examined for this subject.

### Temporal Patterns in the Behavioral data

**Fig 1:**
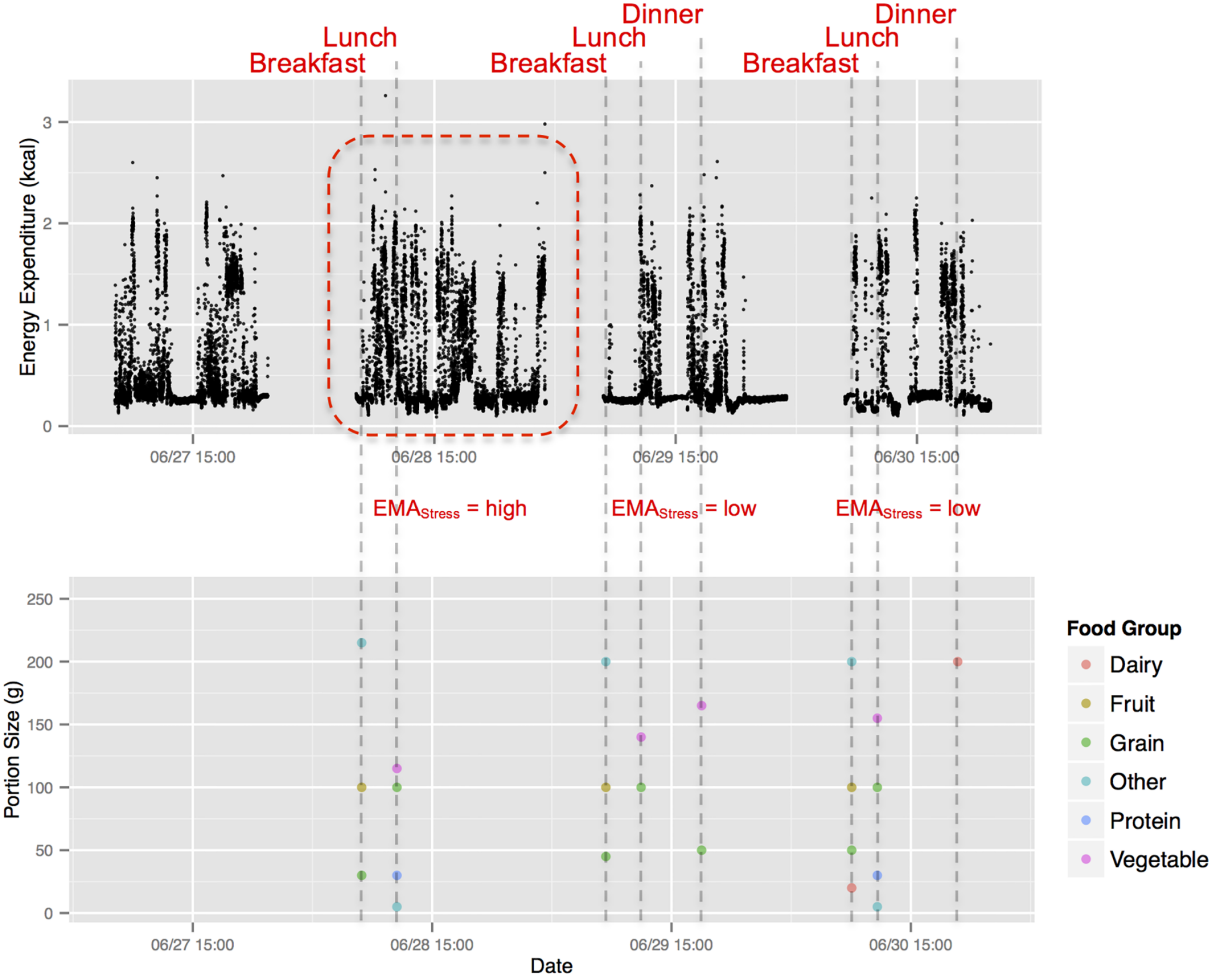
Detailed smartphone-based diet, physical activity, and Ecological Momentary Assessment monitoring data for one subject for four days.

Clear diurnal patterns in both physical activity and diet data were observed in the two weeks of data. As an example, Fig 1 illustrates a detailed time series of diet and energy expenditure data from one of the study participants. The dashed vertical lines indicate the times for breakfast, lunch and dinner meals for three consecutive days, aligned with the corresponding times of physical activity. Two of the 3 days (June 29 and 30, Friday and Saturday) had very similar patterns: a bout of physical activity immediately after breakfast, a period of relatively sedentary activity, followed by a bout of activity that surrounded the lunch meal. After lunch, there was another period of sedentary activity, until mid-afternoon, when there was another somewhat longer duration of physical activity that surrounded the dinner meal. Although June 27^th^ (Wednesday) was not a day in which diet was assessed for this person, the pattern of physical activity was very similar to the two days just described. In contrast, June 28^th^ (Thursday) was markedly different. There was fairly intensive activity throughout the day. While breakfast and lunch meals were recorded as normal, the dinner meal was not. The EMA stress level for this day was higher than that reported on the two following days. While there was a tendency for this person to follow a routine pattern of diet, there was also a tendency for physical activity and emotion to potentially affect this person’s eating behavior.

### Individual-based and Combined Models

**Fig 2:**
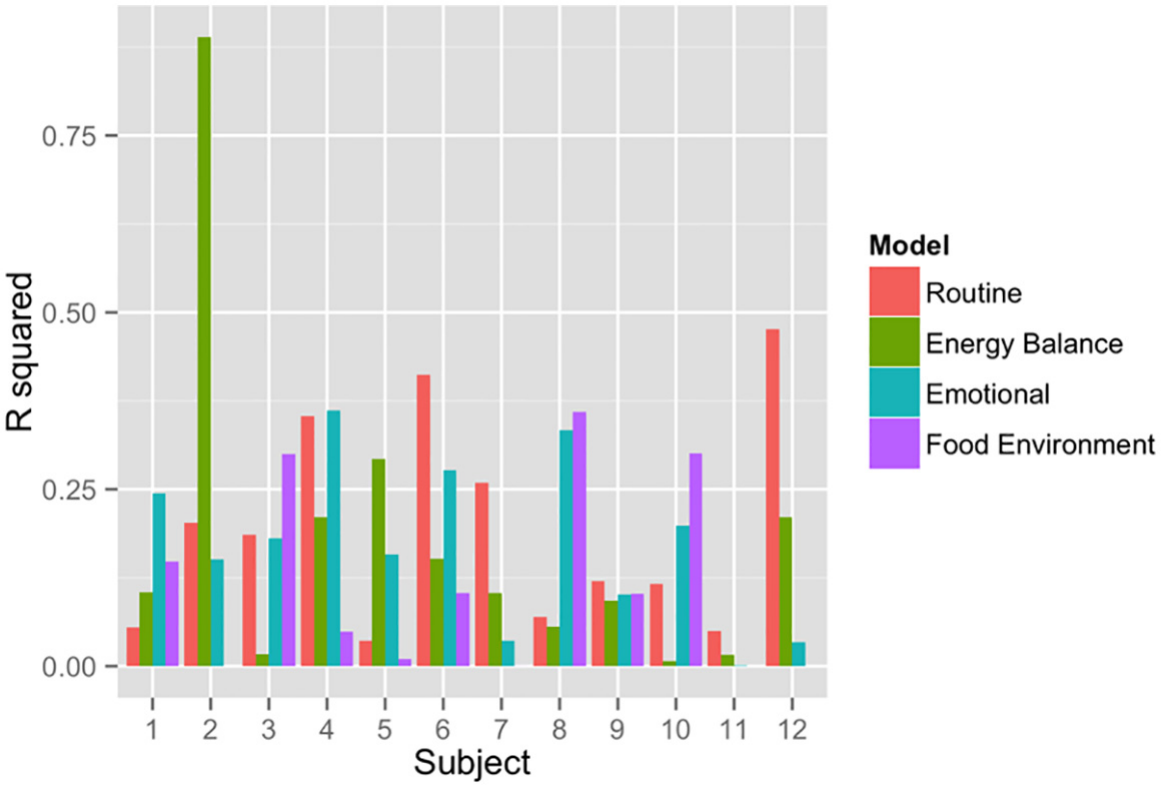
Coefficients of determination for different smartphone-based diet models.

Fig 2 illustrates the R^2^ results for the different typology models applied to each individual subject (See S1 File for details on model estimates). For this cohort, the routine model fit five individuals best. The food environment fit three individuals best. And, energy balance and emotion models each fit two individuals best. The food environment model not fit for Subject 2 because GPS data were not available for this person, perhaps because the participant turned off the GPS in the phone. Subject 2 was markedly different from others in that this person had the worst compliance, recording only 1 week of data. Subject 2 was the only obese subject in our study with a BMI of 30.

For four of the five subjects that the Routine model fit best, breakfast portion sizes tended to be smaller than dinner portions. On average the model estimated between 6.2 and 228 g difference for these subjects’ breakfast and dinner portion sizes. For the remaining fifth individual, we found that lunchtime portion sizes tended to greater (model estimate of 206 g more) than dinnertime portions.

There was support for the influence of the food environment on certain individuals’ diet patterns. The food environment explained a notable larger amount of variation in eating patterns compared to other hypothesized models for subjects 3 and 10. However, in the case of subject 8, the emotion model performed almost as well as the food environment model in explaining eating patterns.

Table 3 lists the coefficients of determination for all typology models applied to each individual. For comparison, the R^2^ is provided for each individual when fit to a model that estimates coefficients across the combined dataset with all individuals. Generally, when any of the models were applied to the combined data, the models tended to fit relatively poorly to the individual compared to individual-based model. In contrast, when certain models were applied to individuals, the coefficients of determinations tended to be quite high. For example, the routine model explained over 47% of the variation in food portion sizes for subject 12, the energy balance model explained over 88% of the variation in portion sizes for subject 2, and the emotional model explained over 36% of the variation in portion sizes for subject 4.

**Table 3:** The Coefficients of Determination for Individual-based models and models with data from all subjects combined.

	Typology Model							
	Routine		Energy Balance		Emotional		Food Environment	
Subject	R^2^	R^2^ Combined Model	R^2^	R^2^ Combined Model	R^2^	R^2^ Combined Model	R^2^	R^2^ Combined Model
1	0.055	<0.001	0.104	<0.001	0.244	<0.001	0.148	<0.001
2	0.203	0.069	0.889	<0.001	0.151	<0.001	-	-
3	0.186	<0.001	0.017	0.002	0.181	<0.001	0.300	<0.001
4	0.353	0.086	0.210	0.004	0.361	<0.001	0.049	0.041
5	0.036	<0.001	0.293	<0.001	0.158	<0.001	0.010	<0.001
6	0.412	0.059	0.152	<0.001	0.277	<0.001	0.103	<0.001
7	0.259	<0.001	0.103	<0.001	0.036	<0.001	<0.001	<0.001
8	0.070	<0.001	0.055	<0.001	0.333	<0.001	0.360	<0.001
9	0.120	0.091	0.093	0.013	0.101	0.004	0.102	<0.001
10	0.117	<0.001	0.007	<0.001	0.198	<0.001	0.300	<0.001
11	0.050	<0.001	0.016	<0.001	0.001	<0.001	<0.001	<0.001
12	0.476	0.142	0.211	<0.001	0.034	<0.001	<0.001	<0.001

There was insufficient monitoring data to fit a “full” multivariate model for each person, with all aspects of routine, energy balance, emotion, and food environment. However, it was possible to fit this multivariate model with data from all individuals combined. The full multivariate model produced an R^2^ of 0.30. Controlling for all other factors (timing, physical activity, and emotion), the only significant predictor was the food environment variable (coefficient 0.32, 95% CI [0.16, 0.49]), indicating a 32% increase in portion sizes per food establishment encountered within 0.25 km of one’s Staypoints.

The mixed effects model, which also controlled for timing, physical activity, and emotion, resulted in a similar coefficient estimate (0.21 95% CI [-0.17, 0.59]) for the food environment, which, however, was not statistically significant. Consistent with our individual-based modeling results, a considerable amount (45.2%) of residual variation was attributed to between subject random effects.

### Comparison of Different Food Environment-based Models

Given the above findings indicating the importance of the food environment for this cohort, we examined the role of the individual food establishment types. We used the aforementioned full multivariate model on combined data from all individuals, substituting food establishments of all types, with specific types of establishments available from the Google Place keywords. The results of this analysis (Table 4) indicated that using all types of food tended to perform relatively well in predicting portion sizes. Only the keywords “café” (R^2^ = 0.31), “meal takeaway” (R^2^ = 0.31), and “restaurant” (R^2^ = 0.31), performed slightly better. The first two are notable, in that they indicate some potential for environments with Western-style establishments to be associated with larger portion sizes. We also note that Google’s “café” category tends to also include noodle shops and small Chinese fast food places. The effect sizes for café and meal takeaway establishments on portion sizes were also large (coefficients of 6.2 and 14.7, respectively).

**Table 4:** Comparison of Food Environment Variables^a^.

Food establishment type	Coefficient	95% CI	R^2^
All	0.323	0.158, 0.488	0.298
Bakery	5.266	1.363, 9.169	0.226
Bar	6.115	2.436, 9.794	0.261
Café	6.162	3.163, 9.160	0.310
Convenience store	1.750	0.526, 2.975	0.235
Food	0.334	0.164, 0.504	0.299
Grocery or supermarket	12.21	5.818, 18.60	0.292
Liquor store	10.30	1.537, 19.06	0.208
Meal delivery	14.39	4.551, 24.23	0.238
Meal takeaway	14.71	7.573, 21.84	0.311
Restaurant	0.453	0.232, 0.673	0.310

Each food environment variable is the number of establishments within 0.25 km of the subject’s Staypoints.

^a^ Full models adjusted for time of meal, physical activity, and emotion scores

## Discussion

To our knowledge, this is the only study that has integrated multiple sensor and self-report measures obtained from smartphones to evaluate different typological models of diet behavior. In our study, we found that individual-based models tended to fit better than group models. The individual-based models allow for better tailoring of coefficients to specific persons. While our study focused on understanding eating behavior, this general approach of fitting models to repeated measures on individuals obtained from personal devices, sensors and apps might have broader uses in predicting patterns of behavior. Moreover, we have shown that by evaluating different typological behavioral models, we can potentially identify factors that are most relevant to an individual’s diet pattern. Being able to predict as well as typify baseline behavior has potential uses for developing tailored interventions, and identifying deviations from baseline behavior over the course of an intervention.

Although we did not monitor many people, our study successfully measured multiple dimensions of individual behavior for each person using our smartphone app. Previous studies have focused on assessing one or two types of behaviors with phones. For example, a number of studies have focused only on tracking physical activity [37], while some have also included diet [38–40]. The addition of more people to this particular study would not necessarily help, as we found that individual-based models outperformed group models. Our intent with individual-based models was not to generalize findings across persons, as is the case with traditional regression approaches. While examining data from students was useful for illustrating the methodology of individual-based modeling, our finding of the importance of the food environment as a diet influence may not be generalizable outside of this particular study cohort.

There were challenges in with this dataset because the observation period was limited to two weeks, and seasonal and episodic changes in subjects’ dietary consumptions and physical activities were not captured. An important direction for future work may be to incorporate longer term monitoring data to look not only at seasonal trends, but shifts in behavioral patterns and influences generally. Longer-term monitoring data would also allow for more sophisticated modeling that incorporate feedback dynamics.

Critical to smartphone-based behavioral assessment is the accuracy of the phone-based measures. We have previously evaluated the accuracy of phone-based accelerometry, and have found our CalFit algorithm to correlate well with measurements made by the Actigraph GT3X accelerometer in free-living studies (correlation coefficient of 0.932) [11]. As for phone-based diet assessments, other researchers have automated the processing of food images with some success [41–44], albeit largely in controlled experimental settings for a limited number of food items. In our study, we used a hybrid approach that mixes self-report, objective recording, and review by trained dietitians. Based on the amount of data collected in our study, our approach is a useful balance between subject burden, objectivity, and accurate measurement, which are typical challenges in diet assessment. Phone-based EMA has been useful in a variety of studies, including studies of emotion in youth [45], mood [16], drug-addiction and post-traumatic stress disorder [46], and sexual risk behavior [47].

Although there are daily variations in individuals’ diets, we found that certain individuals tended to follow diet-related typologies or systematic classifications. Typologies are useful frameworks in clinical medicine and nursing for managing patient variability, and have been applied in various fields to categorize patients into treatment groups. For example, type 2 diabetes patients have been typed into “balanced”, “problematic”, “coasters”, “discouraged”, and “distressed” groups using quantitative methods [48], while patient and caregiver pairs have been typed into “patient oriented”, “caregiver oriented”, “collaboratively oriented”, and “complementarily oriented” group using qualitative methods [49]. Typologies have also been useful in characterizing those who are likely to not adhere to prescribed medication due to “lack of knowledge”, “psychosocial resistance”, or “choice for quality of life” [50]. In each of these examples, there are practical implications associated with these types for patient management, tailoring of interventions, and reducing risk. While typologies have these practical benefits, we recognize that individuals are complex, may not fit into only one classification, or can change over time.

In our study we found that the routine model tended most of our subjects, however, the food environment tended to be most important influence across all subjects. We found that the spatial density of the food environment along one’s Staypoints (places where an individual stayed for more than 10 minutes) was the only variable that was significant in the full model with pooled data from all subjects in explaining eating patterns. We found a positive effect. Specifically, greater access to food establishments was associated with consumption of larger food portions. While one should not generalize from our small sample of students, this finding is consistent with literature from large epidemiologic cohort studies conducted in the U.S. [20, 21]. In the Multi-Ethnic Study of Atherosclerosis (MESA) cohort, living near fast food establishments was found to be associated increased risk of eating fast food as well as decreased odds of eating a healthy diet. Similarly, in the longitudinal Coronary Artery Risk Development in Young Adults (CARDIA) study, neighborhood fast food exposure was also found to be associated with fast food consumption, particularly among low-income and male subjects. Unfortunately, Google Places did not offer “fast food” as a keyword search. We found however, that “café” and “meal takeaway” both performed well, and are related to fast food in the Chinese context, and were associated with larger diet portions.

A potential limitation of our food environment analysis is that it is ecologic in nature. We examined food environment only at the neighborhood-level, and did not gather data on the specific food establishments individuals visited. However, an ecologic approach has merits, not only for understanding macro-level factors that condition behavior, but also for examining individual activity patterns in the context of a combination of micro, meso, and macro environmental influences to inform composite or synergistic interventions to promote physical activity [51]. Moreover, neighborhood-level variables integrate nicely with community-based theories (e.g., theory of defensible space[52], theory of restorative environments [53], theory of behavioral settings [54, 55] and the theory of urban imageability[56]). Given our findings, these ecologic theories may also have relevance to understanding adult dietary behaviors in Chinese urban settings.

The research on food portion sizes may shed light on the potential interplay between macro-level factors and micro-level dietary choices. Based on the review by Ello-Martin, et al. [22], very young children are generally able to self-regulate their food intake based on hunger and satiety cues. However, by 4–5 years, they begin to lose this ability: when presented with larger portions, children tend to consume more. This may be the result of conditioning, as research has shown that children who are rewarded for cleaning their plates tend to consume more [57]. As adults, individuals provided with larger portion sizes also consume more, and yet, report similar levels of fullness after varying sized meals. Although it is unclear how these micro-level findings on portion size extrapolate to macro-level associations between food environment access and consumption, the aforementioned U.S.-based food environment studies, and our findings of a positive association between the macro-level food environment and micro-level portion sizes of diets from this small Chinese cohort, suggest that further research in this area is warranted.

Excluding the routine and ecologic food environment typology, there were slight tendencies for individuals to either fit the energy balance, or emotional models. For those who fit the routine model, further work might consider the use of chronotypes (i.e., “morningness” and “eveningness”) to improve our understanding time-related eating patterns [31, 32, 58]. Also, the theory of behavioral settings [54, 55]–that certain physical settings are associated with recurring patterns—may also lead to further understanding of the interplay between routine eating and place, especially given our findings related to the food environment.

For some individuals, the emotional model performed better than the routine model. The relationship between mood and diet has been explored in both experimental and natural studies. And there are likely feedbacks between mood and diet that we have not fully modeled in our study. Support for this type of model is based on early experimental work that has found that individuals given doses of caffeine report high levels vigor, clarity of mind, energy, etc., while doses of tryptophan tend to increase reports of lethargy and somnolence [59]. Moreover, there is considerable research that has linked negative mood states with a preference for relatively unhealthy foods, and conversely, positive mood with healthy foods [35]. This literature suggests that future work with mobile phone assessments might go beyond quantifying portion size, to characterizing foods as unhealthy/healthy and its relationship to self-reported EMA of emotion.

## Conclusions

In summary, a typological modeling approach can be useful in understanding individual dietary behaviors in our cohort. This approach may be applicable to the study of other human behaviors, particularly those that collect repeated measures on individuals. Smartphones and other personal devices are well suited to providing these repeated measures. The smartphone app and analytic methods are currently being applied to larger cohort studies of physical activity and diet behavior in two regions of China. These larger ongoing studies will be used to validate our findings of the importance of the food environment on Chinese diets, and to quantify the proportions of individuals that follow specific dietary typologies. Our study demonstrated individual-based modeling could be useful, and further research using larger datasets and typological approaches are needed for studying human behaviors.

## Supporting Information

S1 File Model coefficients and confidence intervals. (PDF)

## References

[1] Swan M. Sensor mania! the internet of things, wearable computing, objective metrics, and the quantified self 2.0. Journal of Sensor and Actuator Networks. 2012;1(3):217–53.

[2] Rivera-Pelayo V, Zacharias V, Müller L, Braun S. A framework for applying quantified self approaches to support reflective learning. Mobile Learning. 2012.

[3] McSweeney FK, Murphy ES. The Wiley-Blackwell handbook of operant and classical conditioning [text]. Hoboken: Wiley,; 2014 Available from: Wiley Online Library. Restricted to UC campuses 10.1002/9781118468135.

[4] Bronfenbrenner u. Measuring environment across the life span: emerging methods and concepts. 1st ed Friedman SL, Wachs TD, editors. Washington, DC: American Psychological Association; 1999 xvii, 419 p. p.

[5] Collins LM, Murphy SA, Bierman KL. A conceptual framework for adaptive preventive interventions. Prevention science: the official journal of the Society for Prevention Research. 2004;5(3):185–96. 15470938 10.1023/b:prev.0000037641.26017.00 PMC3544191

[6] Lillie EO, Patay B, Diamant J, Issell B, Topol EJ, Schork NJ. The n-of-1 clinical trial: the ultimate strategy for individualizing medicine? Personalized medicine. 2011;8(2):161–73. 10.2217/pme.11.7 21695041 PMC3118090

[7] Lakerveld J, Glonti K, Rutter H. Individual and contextual correlates of obesity-related behaviours and obesity: the SPOTLIGHT project. Obesity reviews: an official journal of the International Association for the Study of Obesity. 2016;17 Suppl 1:5–8. 10.1111/obr.12384 . 26879108

[8] Kremers SP, de Bruijn GJ, Visscher TL, van Mechelen W, de Vries NK, Brug J. Environmental influences on energy balance-related behaviors: a dual-process view. Int J Behav Nutr Phys Act. 2006;3:9 10.1186/1479-5868-3-9 16700907 PMC1481572

[9] Kumanyika S, Jeffery RW, Morabia A, Ritenbaugh C, Antipatis VJ, Public Health Approaches to the Prevention of Obesity Working Group of the International Obesity Task F. Obesity prevention: the case for action. Int J Obes Relat Metab Disord. 2002;26(3):425–36. 10.1038/sj.ijo.0801938 . 11896500

[10] Lakerveld J, Brug J, Bot S, Teixeira PJ, Rutter H, Woodward E, et al Sustainable prevention of obesity through integrated strategies: The SPOTLIGHT project's conceptual framework and design. BMC Public Health. 2012;12:793 10.1186/1471-2458-12-793 22985293 PMC3490949

[11] Donaire-Gonzalez D, de Nazelle A, Seto E, Mendez M, Nieuwenhuijsen MJ, Jerrett M. Comparison of physical activity measures using mobile phone-based CalFit and Actigraph. J Med Internet Res. 2013;15(6):e111 Epub 2013/07/31. 10.2196/jmir.2470 23896156 PMC3713904

[12] Seto E, Martin E, Yang A, Yan P, Gravina R, Lin I, et al. Opportunistic Strategies for Lightweight Signal Processing for Body Sensor Networks. PETRAE conference, workshop on Light-weight Signal Processing for Computationally Intensive BSN Applications; Samos, Greece2010.

[13] Shiffman S, Stone AA, Hufford MR. Ecological momentary assessment. Annu Rev Clin Psychol. 2008;4:1–32. 18509902 10.1146/annurev.clinpsy.3.022806.091415

[14] Stone AA, Shiffman SS, DeVries MW. Ecological momentary assessment In: Kahneman D, Diener E, Schwarz N, editors. Well-being: The foundations of hedonic psychology. New York, NY, US: Russell Sage Foundation; 1999 p. 26–39.

[15] Jon Noronha EH, Haoqi Zhang, Krzysztof Z. Gajos editor Platemate: crowdsourcing nutritional analysis from food photographs. Proceedings of the 24th annual ACM symposium on User interface software and technology; 2011 October 16–19, 2011; Santa Barbara, California, USA

[16] Courvoisier DS, Eid M, Lischetzke T, Schreiber WH. Psychometric properties of a computerized mobile phone method for assessing mood in daily life. Emotion. 2010;10(1):115 10.1037/a0017813 20141308

[17] Zhu K, Pu B, Xu X-l. Epidemiological survey on simple obesity in 0~ 7 years old children in Kunming city. Chin J Child Health Care. 2008;16(6):696–8.

[18] Qiu H, Zhang M-r, Li Z-k. Study on the Epidemiological Characteristics of Overweight and Obesity in Kunming. Chinese Primary Health Care. 2009;4:037.

[19] Caspi CE, Sorensen G, Subramanian SV, Kawachi I. The local food environment and diet: a systematic review. Health & place. 2012;18(5):1172–87. 10.1016/j.healthplace.2012.05.006 22717379 PMC3684395

[20] Boone-Heinonen J, Gordon-Larsen P, Kiefe CI, Shikany JM, Lewis CE, Popkin BM. Fast food restaurants and food stores: longitudinal associations with diet in young to middle-aged adults: the CARDIA study. Archives of internal medicine. 2011;171(13):1162–70. 10.1001/archinternmed.2011.283 21747011 PMC3178268

[21] Moore LV, Diez Roux AV, Nettleton JA, Jacobs DR, Franco M. Fast-food consumption, diet quality, and neighborhood exposure to fast food: the multi-ethnic study of atherosclerosis. American journal of epidemiology. 2009;170(1):29–36. 10.1093/aje/kwp090 19429879 PMC2733038

[22] Ello-Martin JA, Ledikwe JH, Rolls BJ. The influence of food portion size and energy density on energy intake: implications for weight management. The American journal of clinical nutrition. 2005;82(1 Suppl):236S–41S. . 16002828 10.1093/ajcn/82.1.236S

[23] Ledikwe JH, Ello-Martin JA, Rolls BJ. Portion sizes and the obesity epidemic. The Journal of nutrition. 2005;135(4):905–9. . 15795457 10.1093/jn/135.4.905

[24] Young LR, Nestle M. The contribution of expanding portion sizes to the US obesity epidemic. American journal of public health. 2002;92(2):246–9. 11818300 10.2105/ajph.92.2.246 PMC1447051

[25] Ayala GX. An experimental evaluation of a group- versus computer-based intervention to improve food portion size estimation skills. Health Educ Res. 2006;21(1):133–45. 10.1093/her/cyh049 16100228 PMC3724534

[26] Hernandez T., W L, Kuehn D., Rubotzky K., Moser-Veillon P., Godwin S., Thompson C., Wang C.. Portion size estimation and expectation of accuracy. J Food Comp Anal. 2006;(19 (suppl)):pp. S14–S21.

[27] Godwin SL, Chambers Et, Cleveland L. Accuracy of reporting dietary intake using various portion-size aids in-person and via telephone. J Am Diet Assoc. 2004;104(4):585–94. 10.1016/j.jada.2004.01.006 . 15054344

[28] Williamson DA, Allen HR, Martin PD, Alfonso AJ, Gerald B, Hunt A. Comparison of digital photography to weighed and visual estimation of portion sizes. J Am Diet Assoc. 2003;103(9):1139–45. 10.1053/jada.2003.50567 . 12963941

[29] Lucas F, N M, Villeminot S, Kaaks R, Clavel-Chapelon F. Estimation of food portion size using photographs: Relative validity, strengths, weaknesses and recommendations. J Hum Nutr Diet 1995;8:65–74.

[30] Montoliu R, Gatica-Perez D, editors. Discovering human places of interest from multimodal mobile phone data. Proceedings of the 9th International Conference on Mobile and Ubiquitous Multimedia; 2010: ACM.

[31] Fleig D, Randler C. Association between chronotype and diet in adolescents based on food logs. Eat Behav. 2009;10(2):115–8. 10.1016/J.Eatbeh.2009.03.002 WOS:000275021600006. 19447353

[32] Schubert E, Randler C. Association between chronotype and the constructs of the Three-Factor-Eating-Questionnaire. Appetite. 2008;51(3):501–5. 10.1016/J.Appet.2008.03.018 WOS:000259930900013. 18479778

[33] Thompson DA, Wolfe LA, Eikelboom R. Acute effects of exercise intensity on appetite in young men. Med Sci Sports Exerc. 1988;20(3):222–7. . 3386499 10.1249/00005768-198806000-00002

[34] Verger P, Lanteaume MT, Louis-Sylvestre J. Free food choice after acute exercise in men. Appetite. 1994;22(2):159–64. 10.1006/appe.1994.1015 . 8037440

[35] Christensen L. Effects of eating behavior on mood: a review of the literature. The International journal of eating disorders. 1993;14(2):171–83. . 8401550 10.1002/1098-108x(199309)14:2<171::aid-eat2260140207>3.0.co;2-u

[36] WHO. Global Database on Body Mass Index 2014. Available from: http://apps.who.int/bmi/index.jsp?introPage=intro_3.html.

[37] O'Reilly GA, Spruijt-Metz D. Current mHealth technologies for physical activity assessment and promotion. American journal of preventive medicine. 2013;45(4):501–7. 10.1016/j.amepre.2013.05.012 24050427 PMC4199827

[38] Carter MC, Burley VJ, Nykjaer C, Cade JE. 'My Meal Mate' (MMM): validation of the diet measures captured on a smartphone application to facilitate weight loss. The British journal of nutrition. 2013;109(3):539–46. 10.1017/S0007114512001353 . 22717334

[39] Hebden L, Cook A, van der Ploeg HP, Allman-Farinelli M. Development of smartphone applications for nutrition and physical activity behavior change. JMIR research protocols. 2012;1(2):e9 10.2196/resprot.2205 23611892 PMC3626164

[40] Hughes DC, Andrew A, Denning T, Hurvitz P, Lester J, Beresford S, et al BALANCE (Bioengineering Approaches for Lifestyle Activity and Nutrition Continuous Engagement): developing new technology for monitoring energy balance in real time. Journal of diabetes science and technology. 2010;4(2):429–34. 20307404 10.1177/193229681000400224 PMC2864179

[41] Six BL, Schap TE, Zhu FM, Mariappan A, Bosch M, Delp EJ, et al Evidence-based development of a mobile telephone food record. Journal of the American Dietetic Association. 2010;110(1):74–9. 10.1016/j.jada.2009.10.010 20102830 PMC3042797

[42] Zhu F, Bosch M, Woo I, Kim S, Boushey CJ, Ebert DS, et al The use of mobile devices in aiding dietary assessment and evaluation. Selected Topics in Signal Processing, IEEE Journal of. 2010;4(4):756–66. 10.1109/JSTSP.2010.2051471 PMC2941896 20862266

[43] Kong F, Tan J. DietCam: Automatic dietary assessment with mobile camera phones. Pervasive and Mobile Computing. 2012;8(1):147–63.

[44] Woo I, Ostmo K, Kim S, Ebert DS, Delp EJ, Boushey CJ. Automatic portion estimation and visual refinement in mobile dietary assessment. Computational Imaging. 2010;8(7533):1. 10.1117/12.849051 PMC3254118 22242198

[45] Silk JS, Forbes EE, Whalen DJ, Jakubcak JL, Thompson WK, Ryan ND, et al Daily emotional dynamics in depressed youth: A cell phone ecological momentary assessment study. Journal of experimental child psychology. 2011;110(2):241–57. 10.1016/j.jecp.2010.10.007 21112595 PMC3061240

[46] Fletcher RR, Tam S, Omojola O, Redemske R, Fedor S, Moshoka JM, editors. Mobile application and wearable sensors for use in cognitive behavioral therapy for drug addiction and PTSD. Pervasive Computing Technologies for Healthcare (PervasiveHealth), 2011 5th International Conference on; 2011: IEEE. 10.1109/IEMBS.2011.6090513 22254678

[47] Hensel DJ, Fortenberry JD, Harezlak J, Craig D. The feasibility of cell phone based electronic diaries for STI/HIV research. BMC medical research methodology. 2012;12(1):75. 22691189 10.1186/1471-2288-12-75 PMC3480871

[48] Fisher L, Chesla CA, Skaff MA, Gilliss C, Kanter RA, Lutz CP, et al Disease management status: a typology of Latino and Euro-American patients with type 2 diabetes. Behavioral medicine. 2000;26(2):53–66. 10.1080/08964280009595752 . 11147290

[49] Buck HG, Kitko L, Hupcey JE. Dyadic heart failure care types: qualitative evidence for a novel typology. The Journal of cardiovascular nursing. 2013;28(6):E37–46. 10.1097/JCN.0b013e31827fcc4c 23388704 PMC3655126

[50] Shaw RJ, Palmer L, Blasey C, Sarwal M. A typology of non-adherence in pediatric renal transplant recipients. Pediatric transplantation. 2003;7(6):489–93. . 14870900 10.1046/j.1397-3142.2003.00117.x

[51] King AC, Stokols D, Talen E, Brassington GS, Killingsworth R. Theoretical approaches to the promotion of physical activity: forging a transdisciplinary paradigm. American journal of preventive medicine. 2002;23(2 Suppl):15–25. . 12133734 10.1016/s0749-3797(02)00470-1

[52] Newman O. Defensible space; crime prevention through urban design. New York,: Macmillan; 1972 xvii, 264 p. p.

[53] Kaplan S. The Restorative Benefits of Nature—toward an Integrative Framework. J Environ Psychol. 1995;15(3):169–82. 10.1016/0272-4944(95)90001-2 WOS:A1995TC98400002.

[54] Barker RG. Ecological psychology; concepts and methods for studying the environment of human behavior. Stanford, Calif.,: Stanford University Press; 1968 vi, 242 p. p.

[55] Schoggen P, Barker RG, Fox KA. Behavior settings: a revision and extension of Roger G. Barker's Ecological psychology. Stanford, Calif.: Stanford University Press; 1989 xii, 419 p.

[56] Lynch K. The image of the city. Cambridge Mass.: Technology Press; 1960 194 p. p.

[57] Birch LL, Mcphee L, Shoba BC, Steinberg L, Krehbiel R. Clean up Your Plate—Effects of Child Feeding Practices on the Conditioning of Meal Size. Learn Motiv. 1987;18(3):301–17. 10.1016/0023-9690(87)90017-8 WOS:A1987J858100005.

[58] Gruber R, Wiebe S, Coffey EB, Elgie B, Frenette S, Robert M, et al The Association between Chronotype and Sleep Patterns in Children. Sleep. 2010;33:A80–A. WOS:000208208000232.

[59] Leathwood PD, Pollet P. Diet-induced mood changes in normal populations. Journal of psychiatric research. 1982;17(2):147–54. . 6764931 10.1016/0022-3956(82)90016-4

