# Supplementary material for: Models of Individual Dietary Behavior Based on Smartphone Data: The Influence of Routine, Physical Activity, Emotion, and Food Environment
Source: PLoS One. 2016 Apr 6;11(4):e0153085. doi: 10.1371/journal.pone.0153085 (PMC4822823; doi:10.1371/journal.pone.0153085)
Supplement: S1 File — (PDF) [file pone.0153085.s001.pdf]

S1 File. Model coefficients and confidence intervals

Table 1. Routine model coefficients and confidence intervals

**Routine Models**				
	**Variable Name**	**Estimate**	**95% CI**	
Combined data all subjects	Intercept	302.89	265.74	340.03
	Breakfast indicator	-67.84	-123.46	-12.22
	Lunch indicator	6.2	-48.79	61.18
Individual 1	Intercept	241.4	146.78	336.02
	Breakfast indicator	-32.83	-156.72	91.06
	Lunch indicator	17.77	-110.35	145.88
Individual 2	Intercept	423.33	236.89	609.78
	Breakfast indicator	-177.9	-400.75	44.94
	Lunch indicator	-111.33	-347.17	124.5
Individual 3	Intercept	151.67	0.09	303.25
	Breakfast indicator	176.19	-30.38	382.76
	Lunch indicator	148.33	-66.03	362.7
Individual 4	Intercept	445	294.99	595.01
	Breakfast indicator	-256	-478.5	-33.5
	Lunch indicator	-204.8	-427.3	17.7
Individual 5	Intercept	508.83	301.55	716.12
	Breakfast indicator	-100.71	-374.92	173.5
	Lunch indicator	-77.67	-370.81	215.48
Individual 6	Intercept	363.33	243.12	483.55
	Breakfast indicator	-228.33	-445.05	-11.62
	Lunch indicator	137.67	-63.49	338.82
Individual 7	Intercept	193.8	107.01	280.59
	Breakfast indicator	-108.8	-271.17	53.57
	Lunch indicator	29.77	-83.86	143.4
Individual 8	Intercept	220	122.21	317.79
	Breakfast indicator	62.5	-166.83	291.83
	Lunch indicator	70	-84.61	224.61

Individual 9	Intercept	299.09	214.9	383.28
	Breakfast indicator	-111.09	-261.69	39.51
	Lunch indicator	1.62	-133.38	136.62
Individual 10	Intercept	253.75	91.89	415.61
	Breakfast indicator	-123.75	-321.99	74.49
	Lunch indicator	-38.75	-241.66	164.16
Individual 11	Intercept	333.71	232.64	434.79
	Breakfast indicator	-6.21	-173.83	161.4
	Lunch indicator	-98.71	-283.25	85.82
Individual 12	Intercept	227.22	131.99	322.46
	Breakfast indicator	-99.37	-243.35	44.62
	Lunch indicator	205.63	61.65	349.62

Table 2. Energy balance model coefficients and confidence intervals

**Energy Balance Models**				
	**Variable Name**	**Estimate**	**95% CI**	
Combined data all subjects	Intercept	300.83	228.34	373.33
	kcal same hr	-17.05	-96.74	62.65
	kcal average of 3 hrs before	-16.7	-125.65	92.25
Individual 1	Intercept	110.48	-96.74	317.7
	kcal same hr	83.43	-146.29	313.14
	kcal average of 3 hrs before	168.06	-143.68	479.79
Individual 2	Intercept	-503.83	-1096.2	88.53
	kcal same hr	727.83	255.11	1200.55
	kcal average of 3 hrs before	220.2	-255.73	696.14
Individual 3	Intercept	345.84	-30.89	722.57
	kcal same hr	-23.51	-343.88	296.85
	kcal average of 3 hrs before	-112.78	-591.78	366.22
Individual 4	Intercept	282.53	-113.27	678.32
	kcal same hr	-191.24	-565.41	182.93
	kcal average of 3 hrs before	248.41	-254.13	750.96

Individual 5	Intercept	118.17	-174.79	411.13
	kcal same hr	528.41	-141.75	1198.57
	kcal average of 3 hrs before	374.11	11.67	736.56
Individual 6	Intercept	69.08	-341.73	479.88
	kcal same hr	75.22	-268.06	418.5
	kcal average of 3 hrs before	577.41	-180.07	1334.89
Individual 7	Intercept	181.76	14.84	348.68
	kcal same hr	116.39	-120.19	352.98
	kcal average of 3 hrs before	-135.25	-513.25	242.75
Individual 8	Intercept	343.92	81.21	606.63
	kcal same hr	-17.76	-324.47	288.95
	kcal average of 3 hrs before	-128.69	-459.75	202.36
Individual 9	Intercept	320.37	147.26	493.49
	kcal same hr	56.1	-246.38	358.58
	kcal average of 3 hrs before	-152.53	-383.33	78.27
Individual 10	Intercept	211.41	-63.29	486.1
	kcal same hr	-39.67	-289.11	209.77
	kcal average of 3 hrs before	11.54	-453.17	476.24
Individual 11	Intercept	278.61	-22.77	580
	kcal same hr	84.62	-209.91	379.14
	kcal average of 3 hrs before	-58.43	-660.96	544.1
Individual 12	Intercept	174.48	-110.7	459.65
	kcal same hr	196.06	-12.88	405.01
	kcal average of 3 hrs before	-154.4	-470.88	162.08

Table 3. Emotion model coefficients and confidence intervals

**Emotion Models**				
	**Variable Name**	**Estimate**	**95% CI**	
Combined data all subjects	Intercept	281.84	257.95	305.74
	PC1 (happiness)	-5.81	-24.03	12.42
	PC2 (tiredness)	-1.04	-27.42	25.34
Individual 1	Intercept	267.49	209.93	325.06

	PC1 (happiness)	-95.37	-187.67	-3.06
	PC2 (tiredness)	73.61	-32.88	180.1
Individual 2	Intercept	-922.45	-4855.6	3010.7
	PC1 (happiness)	972.59	-2232.21	4177.4
	PC2 (tiredness)	1416.61	NA	NA
Individual 3	Intercept	262.1	83.69	440.5
	PC1 (happiness)	21.71	-143.68	187.11
	PC2 (tiredness)	276.39	-58.84	611.63
Individual 4	Intercept	-81.68	-420.57	257.2
	PC1 (happiness)	319.79	50.58	589
	PC2 (tiredness)	-19.65	-235.02	195.73
Individual 5	Intercept	553.21	325.38	781.04
	PC1 (happiness)	94.57	-126.59	315.74
	PC2 (tiredness)	81.74	-41.24	204.72
Individual 6	Intercept	350.39	234.66	466.12
	PC1 (happiness)	-29.62	-246.48	187.23
	PC2 (tiredness)	-380.49	-720.13	-40.86
Individual 7	Intercept	171.91	39.31	304.51
	PC1 (happiness)	-45.12	-216.43	126.2
	PC2 (tiredness)	-31.78	-182.8	119.25
Individual 8	Intercept	138.65	28.27	249.02
	PC1 (happiness)	-81.64	-167.95	4.66
	PC2 (tiredness)	-5.9	-92.81	81
Individual 9	Intercept	224.29	113.23	335.35
	PC1 (happiness)	25.78	-118.78	170.34
	PC2 (tiredness)	62.31	-44.84	169.46
Individual 10	Intercept	203.48	129.59	277.37
	PC1 (happiness)	48.85	-4.15	101.84
	PC2 (tiredness)	-98.84	-307.72	110.04
Individual 11	Intercept	298.44	56.36	540.52
	PC1 (happiness)	-7.1	-178.29	164.09

	PC2 (tiredness)	1.15	-119.53	121.84
Individual 12	Intercept	392.95	-1270.34	2056.25
	PC1 (happiness)	-84.94	-948.24	778.37
	PC2 (tiredness)	3.49	-649.79	656.78

Table 4. Food environment model coefficients and confidence intervals

**Food Environment Models**				
	**Variable Name**	**Estimate**	**95% CI**	
Combined data all subjects	Intercept	180.89	129.18	232.61
	# food establishments within 0.25km	0.31	0.15	0.46
Individual 1	Intercept	68.57	-192.4	329.53
	# food establishments within 0.25km	0.49	-0.46	1.43
Individual 3	Intercept	-357.96	-1217.08	501.16
	# food establishments within 0.25km	1.3	-0.47	3.07
Individual 4	Intercept	187.47	35.81	339.13
	# food establishments within 0.25km	0.22	-0.74	1.18
Individual 5	Intercept	624.52	-693.99	1943.04
	# food establishments within 0.25km	-0.27	-2.74	2.19
Individual 6	Intercept	289.99	-802.37	1382.34
	# food establishments within 0.25km	0.8	-2.46	4.06
Individual 7	Intercept	168.25	-191.84	528.34
	# food establishments within 0.25km	0.29	-33.14	33.73
Individual 8	Intercept	401.71	-41.59	845.01
	# food establishments within 0.25km	-0.39	-1.1	0.33
Individual 9	Intercept	350.44	53.68	647.2
	# food establishments within 0.25km	-0.92	-3.63	1.8
Individual 10	Intercept	-27.14	-228.14	173.85
	# food establishments within 0.25km	82.86	-20.25	185.96
Individual 11	Intercept	217.5	11.1	423.9

	# food establishments within 0.25km	64.86	NA	NA
Individual 12	Intercept	138.33	11.07	265.6
	# food establishments within 0.25km	49.51	NA	NA

Table 5. Full model coefficients and confidence intervals

**Full Model**				
	**Variable Name**	**Estimate**	**95% CI**	
	Intercept	272.42	152.42	392.43
	Breakfast indicator	-57.45	-150.08	35.17
	Lunch indicator	56.89	-33.46	147.23
	kcal same hr	-88.92	-217.28	39.44
	kcal average of 3 hrs before	-67.44	-223.37	88.49
	PC1 (happiness)	1.05	-32.31	34.41
	PC2 (tiredness)	4.78	-58.64	68.21
	# food establishments within 0.25km	0.32	0.16	0.49
